# Supplementary material for: Are carbon emissions trading and green financial instruments synergistic? -Comprehensive quantitative research based on content analysis
Source: PLoS One. 2024 Mar 7;19(3):e0298601. doi: 10.1371/journal.pone.0298601 (PMC10919601; doi:10.1371/journal.pone.0298601)
Supplement: S1 Data — (ZIP) [file pone.0298601.s001.zip › Data/地级市Z_F工作报告.zip!/╔┬╬≈/═¡┤¿╩╨/└┤╫╘╣1⁄2╓┌║┼í╛┬φ┐╦╩2╛▌═°í┐_2014.docx]

各位代表：

　　我代表市人民政府向大会报告工作，请予审议。请市政协委员和列席人员提出意见。

　　一、市十五届人大四次会议确定的目标任务完成情况

　　2013年，面对错综复杂的经济形势，在省委、省政府和市委的坚强领导下，全市人民深入贯彻落实党的十八大和十八届二中、三中全会精神，坚持稳中求进的工作总基调，咬定目标不放松，推动转型不动摇，深化改革不停步，较好地完成了市十五届人大四次会议确定的目标任务。

　　（一）经济持续稳定增长。在传统支柱产业受到冲击、经济下行压力较大的情况下，着力稳增长、调结构、促改革，全市经济持续向好。全年实现生产总值321.98亿元，增长13.8%，增速全省第二；新增规模企业23户，规模以上工业实现增加值183.09亿元，增长18.7%，高于全省5.6个百分点；地方财政收入24.1亿元，同口径增长21.7%，增速全省第四；城镇居民人均可支配收入24495元，增长11.7%，增速全省第二；农民人均纯收入8140元，增长14.1%，增速全省第五。

　　（二）项目建设有力推进。全社会固定资产投资完成260.02亿元，增长28%，增速全省第一。100个市级重点项目完成投资165.29亿元，23个项目建成投运，30个项目开工建设。延西高速公路新区至何家坊段建成通车。我市入选全国节能减排财政政策综合示范城市。

　　（三）工业不断优化升级。14处停产整顿煤矿经省上批复开工建设，白石崖、秀房沟、铜鑫煤矿机械化改造后联合试运转，照金煤矿产能达到180万吨。淘汰落后水泥产能385万吨、电解铝产能9万吨。大秦铝业建成投产，30万吨碳素项目建成试生产。煤、电、铝、水泥实现增加值154.52亿元，增长16.7%。陕煤化PVC管材、西安重装机械、陕汽零部件加工二期等项目建成投产，接续替代产业实现增加值8.03亿元，增长9.6%。宜君石油资源开发完成投资13.5亿元，形成10万吨原油产能。

　　（四）服务业发展势头良好。与陕文投、陕煤化合作，用一年时间建成照金红色旅游名镇一期工程。宜君荣获“最美中国”自然生态旅游目的地称号。全年接待游客数量、旅游总收入分别增长30.6%、42.8%。编制了中医药产业园区规划，组建健康产业投资公司。社会消费品零售总额72.36亿元，增长15.9%，增速全省第一。外贸进出口总额1629.8万美元，增长31.3%。

　　（五）可持续发展能力不断增强。8个工业园区入驻企业152户，产值占全市工业的61.5%。董家河循环经济产业园被确定为国家循环经济教育示范基地。18个农业园区汇聚龙头企业29户，伊利万头奶牛养殖项目一期即将建成。铜川大樱桃、耀州瓷、铜川苹果获得国家地理标志证明商标，宜君核桃被列入国家地理标志保护产品。全市金融生态环境由C级提升到B级。全年招商引资签约项目164个，153个项目进入实施阶段，到位资金179亿元，增长35.4%，增速全省第二。非公经济增加值占生产总值的48.1%。

　　（六）城市综合服务功能有所提升。新建、改造城市道路6.4公里，整修行车道、人行道3.3万平方米，新增公共绿地61万平方米。开展“清洁铜川”行动，拆除危旧建筑19.2万平方米。宜君县创建为国家卫生县城。黄堡重点示范镇累计完成投资5.8亿元。农村居民进城落户1.6万人。城镇化率61.25%。

　　（七）生态环境持续改善。改造提升20家重点监管企业治污设施，关闭拆除8家水泥企业、62家石碴厂、79个白灰窑和84个坩土窑。实施210国道铜川城市段、药王山景区和坡头工业园区环境综合整治。拆除燃煤锅炉78台，新建燃气锅炉98台。新增天然气用户1.37万户，城市气化率75%。城市空气质量优良天数330天。万元生产总值能耗下降5.31%，二氧化硫、氮氧化物、化学需氧量、氨氮排放，分别削减5.11%、14.31%、2.21%、6.12%，均超额完成年度任务。荣获全国绿化模范城市、宜居生态示范城市称号。野化放飞的朱鹮生存状态良好。

　　（八）民生工作力度加大。全年财政一般预算支出的80.6%、新增财力的81.3%用于民生。城镇新增就业1.88万人，登记失业率3.45%。开工保障房8648套，竣工1.3万套，2.6万人喜迁新居。我市荣获全省保障性安居工程建设二等奖，印台区评为十佳区县。搬迁安置地质灾害点群众1003户3646人。城乡居民社会养老保险参保率98.77%，城镇基本医疗保险参保率、新农合参合率分别达到98.79%、99.04%。保障城乡低保对象9.03万人，实施医疗救助3.69万人次，2.04万农村贫困人口脱贫。实施了“一元民生保险”、城乡居民社会养老保险丧葬费补贴、计生特殊家庭补助，开展财政惠民补贴资金“一卡通”兑付改革。建成农村幸福院122个。提高高龄老人生活保健补贴标准，为5200名残疾人免费适配辅助器具。建成20个城市社区服务体系项目、14个农村社区服务中心。实现乡镇公办中心幼儿园全覆盖。实施农村义务教育营养改善计划，2.26万名学生受益。图书馆公共服务一体化示范项目通过国家验收。建成60个行政村、20个社区文化活动室和24个村级农民体育健身工程，安装广播电视户户通接收设备2万套。信访工作综合考核全省排名第一，连续三年实现“三无”区县全覆盖。安全生产形势平稳。社会治安状况良好，耀州区2013年度社会治安状况满意率在全省29个城市区和县级市中排名第二。

　　（九）政府自身建设进一步加强。修订了《市政府工作规则》。办理人大代表建议57件、政协提案291件，代表委员满意率99.7%。办理《人民网》留言148件、“市长信箱”1325件。精简市级行政审批事项34项。开展公民代表进政府、大学生暑期到政府机关见习活动，在新浪、新华和腾讯网同步开通“铜川发布”政务微博，邀请公民代表列席市政府全体会、常务会议。清理规范办公用房和公务用车，市本级“三公”经费下降12.6%。开展“庸懒散”专项整治，政府机关作风有所好转。

　　双拥共建活动深入开展，妇女儿童事业加快发展，民族宗教、审计、统计、档案、人防、气象、防震减灾等工作都取得了新成绩。

　　各位代表，过去一年的成绩来之不易。是市委统揽全局、科学决策的结果，是市人大、市政协和社会各界支持监督的结果，更是全市人民团结拼搏、真抓实干的结果。我代表市政府，向全市人民，向关心支持铜川发展的社会各界人士，表示衷心的感谢！

　　同时，我们也清醒认识到，发展中仍存在一些困难和问题，政府工作还有不少缺点和不足。主要是：影响经济增长的结构性问题比较突出，“煤主沉浮”特征明显，接续替代产业处于培育阶段，消费仍是经济发展的短板；创新水平低，人才、资金等要素集聚能力弱，大项目特别是骨干财源项目少，经济增长的内生动力不足；城市规划的刚性约束和建设的系统性有待加强，管理机制和综合配套功能不健全，优化投资环境、城市精细化管理力度还需加大；安全生产基础薄弱，促进群众增收、治污降霾、节能减排、社会治理创新任务艰巨；政府职能转变不到位，一些政府工作人员改革创新能力和服务意识不强。这些，都亟待在今后工作中认真加以解决。

　　二、2014年工作的基本思路和预期目标

　　今年，我们面临的形势依然错综复杂，有利条件和不利因素并存。全国经济正处于结构调整阵痛期、增长速度换挡期，经济下行压力依然较大，但长期向好的基本面没有改变，仍处于大有作为的重要战略机遇期。中央全面深化改革、加快推进新型城镇化、向西开放、共建丝绸之路经济带等重大战略部署，将进一步激活经济发展的内生动力。国务院发布《全国资源型城市可持续发展规划（2013－2020年）》，我市入选全国节能减排财政政策综合示范城市，将获得中省的有力支持。经过多年的转型发展，投资环境不断改善，区县、园区、景区竞相发展的格局正在形成，干事创业的氛围、干部群众的干劲和作风都在一天天向好。这些，必将汇聚成推动转型发展的强大正能量！

　　今年政府工作的总体要求是：以邓小平理论、“三个代表”重要思想、科学发展观为指导，全面贯彻落实党的十八大、十八届二中、三中全会和习近平总书记系列重要讲话精神，贯彻落实省委、省政府及市委的决策部署，以建设经济强、文化兴、生态美的全国知名休闲养生城市为目标，坚持稳中求进工作总基调，把改革创新贯穿于经济社会发展各个领域各个环节，统筹推进新型工业化、信息化、城镇化和农业现代化，全面深化改革扩大开放，强化创新驱动，加快转方式调结构促升级，着力保障和改善民生，切实提高发展质量和效益，实现经济持续健康发展、社会和谐稳定。

　　主要预期目标是：生产总值增长11.5%，地方财政收入同口径增长14%，全社会固定资产投资增长26%，社会消费品零售总额增长15.5%，城镇登记失业率控制在4.5%以内，城乡居民人均可支配收入分别增长11%、13%，居民消费价格涨幅控制在4%左右，人口自然增长率控制在5.5‰以内，万元生产总值能耗下降3%，二氧化硫、氮氧化物、化学需氧量、氨氮排放分别削减0.3%、6%、1%和2%。

　　三、2014年重点工作

　　做好今年政府工作，要以深化改革为动力，以转型发展为统揽，以优化环境为保障, 以改善民生为目的，统筹兼顾，突出重点，务求实效。

　　（一）突出抓好项目建设，夯实持续发展的基础

　　加快重点项目建设。大力实施项目带动战略，扩大投量、加快投速、提升投效。对投资亿元以上、带动转型发展的40个骨干项目，集中力量予以推进。铭帝铝型材一期、金茂国际大厦等26个项目建成投产，旺旺食品三期、深圳美佳华商业广场等42个项目开工建设。延西高速公路何家坊至烈桥立交段年内贯通，加快合凤高速公路关庄至照金段、210国道川口至耀州段改扩建工程建设，开工建设耀州至照金红色旅游公路环线。建设龙潭水库大坝主体工程。

　　抓好招商引资工作。围绕主导产业和延长产业链，瞄准“国字头”、“中字号”企业和知名民企，有针对性地定向招商、专题招商。加强专业招商人才队伍建设，在招大引强上力求取得突破。全年利用市域外资金100亿元以上。

　　着力优化投资环境。实行重点项目服务保障推进制、驻工地专员制、媒体公示制、要素保障制、建设环境保障制、诚信履约制、目标责任考核奖惩制，依法有序推进重点项目建设。落实重点项目金融扶持政策，对当年获得市域内金融机构中长期贷款的重点项目，市财政给予一年贴息扶持。对重点项目建设实行一月一检查、一季一通报、年终考核奖惩，以服务环境的大变化，助推项目建设的“加速度”。

　　（二）加快产业结构调整，构建多元产业体系

　　实施工业强市战略。抓住转型和节能减排示范的机遇，坚决向粗放的生产方式告别，走新型工业化道路。加大煤矿主力矿井安全改造力度，促进新旧矿山有序接替。积极应用清洁利用技术，加快煤炭就地转化。以铝型材、铝合金为重点，加快铝加工项目建设。做精工艺陶瓷，积极发展建筑陶瓷、日用陶瓷。改造提升现有水泥产业，加快发展节能环保型建材。大力发展接续替代产业，扩大煤矿机械、汽车零部件等制造业规模，积极培育新材料、食品、医药健康产业，加快油气、油页岩等资源开发。把工业园区作为新型工业化的主战场，明晰各个园区功能定位，突出主导产业发展，加大基础设施投入，实施人才带动战略，增强园区吸引力和产业承载能力。加强对重点行业和骨干企业的服务，抓好稳产扩销提效。全市规模以上工业增加值增长16%。

　　加快发展现代服务业。放大照金效应，搞好红色培训，扩大对外影响。深化与陕文投、陕煤化等大企业的合作，启动药王山大景区和照金香山二期工程建设，加快大唐养生园和玉华宫、陈炉古镇建设。加快培育养老、健康服务业。伴随着城市转型和空气质量的好转，不断加大财政投入，引导大量社会资本投向旅游业，打造旅游经济新亮点。全年旅游总收入增长20%。挖掘文化资源，创新体制机制，积极培育市场主体，加快文化产业发展。加强文物保护，推动文化与旅游、体育等产业融合。文化产业增加值增长29%。加快商贸服务业发展，提升商业街区、购物商场、连锁酒店的规模和档次，开展餐饮名店、特色小吃店、特色地产品评选推广工作。抓好万村千乡、农超对接、放心粮油全覆盖等工程。积极发展电子商务等新型业态。全年服务业增加值增长8.5%。

　　大力发展现代农业。从数量和质量上坚守耕地红线，完成永久基本农田划定工作。新修改造基本农田2万亩，发展节水灌溉2万亩，耕地保有量140.55万亩，基本农田保护面积不低于111万亩，粮食总产稳定在20万吨以上。加快推进18个省市级现代农业园区建设，启动耀州现代农业示范区建设。建设3个万亩、20个千亩现代果业示范园，新建百亩设施蔬菜示范基地2个。抓好全省奶牛生产示范市建设，启动5个500头标准化奶牛场建设。改造提升3个万头生猪养殖场。建设农产品质量安全追溯体系和市县联网的畜牧生产安全监管服务系统，将农产品质量安全纳入区县、乡镇政府绩效考核范围。

　　（三）以人为核心，全面推进新型城镇化

　　完善新型城镇化规划。以人为本、四化同步、优化布局、生态文明、传承文化，突出地域特色，推进新型城镇化建设。坚持城市、县城、重点镇、新型社区四位一体，通过南拓西展、北疏中控，构筑“北市区—黄堡—董家河—耀州—新区—坡头”的带状组团型中心城区空间格局，带动宜君县城、7个重点镇、50个新型社区、100个中心村统筹协调发展。强化规划的权威性，一张蓝图干到底。

　　提高城市建设水平。加快新区建设，突出形态开发和功能开发。强化产业支撑，推进商业综合体建设。加快华原西道、九州东道、华夏南道建设，实施正阳路、朝阳路地下综合管廊工程，从停车场、公厕、绿化、建筑小品等元素抓起，尽快完善新区综合服务功能。加快耀州城区改造，推进新耀一体化。完善北市区市政设施，加快重点片区改造步伐。支持宜君秀美山城建设。抓好黄堡重点示范镇和照金、陈炉文化旅游名镇建设。启动20个美丽乡村建设。以漆水河、沮河、赵氏河等城市生态组团为纽带，扩大水面和湿地面积，让城市融入自然。城市化归根结底要体现在人的生活质量、水平和环境的改善上。

　　加强城市精细化管理。全面加强市容环境综合整治，全方位探索网格化管理，在规范、系统、精细上持续作为。加快数字城管建设，规范城市管理联合执法。实施城市主干线、出入口连接线、道路节点等区域的美化亮化。坚持徒步检查，强化现场管理。抓好新区公交客运枢纽站场建设，方便市民低碳出行。深化品牌公路、品牌客运创建活动，加强路域环境综合治理。提高市民素质，促进文明卫生习惯的养成。如期建成国家卫生城市。

　　推进农业转移人口市民化。全年实现农村居民进城落户1.6万人。搞好耀州区统筹城乡发展试点，推进城镇基本公共服务常住人口全覆盖。逐步将农民工子女纳入义务教育经费保障和普惠性学前教育、免费中等职业教育范围，使农业转移人口和城镇居民共建共享城市现代文明。

　　（四）加强生态文明建设，努力建设大美铜川

　　出重拳强化污染防治。深化省级环保模范城市创建活动，扎实推进“治污降霾·保卫蓝天”行动，坚决向污染宣战。削减燃煤76万吨，拆除燃煤锅炉125台，淘汰“黄标车”和老旧机动车1500辆。建立严重污染天气应急预案，落实污染排放超过环境承载力的开发建设限制性措施。及时发布空气质量实时信息，向社会公布铜川在全国161个重点城市中的排名情况。按照新标准，全年空气质量优良天数不少于140天。实施污水处理厂提标改造工程，提高中水回用率。推进排污权交易试点。强化污染源在线监控，严厉打击各类环境违法行为。实施农村环境连片综合治理，严禁落后产能和污染企业向我市特别是农村转移。

　　节约集约利用资源。扎实做好节能减排工作，注重新设备、新技术、新工艺运用，全面推进工业、交通、建筑和公共机构节能。实行最严格水资源管理制度，加强饮用水源地保护，开展城乡居民饮用水提质改造专项行动，按照新的标准开展饮用水质量监测，并向社会公布。解决1.5万农村人口的饮水安全问题。建立用水总量控制、用水效率控制和重要水功能区达标率控制指标体系，万元工业增加值用水量较上年下降0.5%。抓好城乡建设用地增减挂钩和工矿废弃地复垦利用工作，依法处理闲置土地。

　　加强生态环境建设。巩固提升全国绿化模范城市创建成果，大力实施绿化铜川五大工程，做好废弃矿山生态环境恢复治理。植树造林12万亩，治理水土流失150平方公里。新增公共绿地60万平方米。干部离任要审“绿色责任”。

　　各位代表，环境保护关系群众切身利益，关乎铜川长远发展，我们按照源头严防、过程严管和后果严惩的思路，铁面执法、铁腕治污，呵护好我们赖以生存的共同家园！

　　（五）加大投入，做细做实改善民生各项工作

　　推动实现更高质量就业。加强技能培训和就业指导，做好高校毕业生、农村转移劳动力、城镇困难人员、退役军人等就业工作，统筹做好淘汰落后产能职工安置和再就业工作。在新区建设大学生创业孵化基地，在黄堡工业园区建设困难企业职工再就业综合示范基地，在照金镇建设农民就业创业示范基地。全年发放小额担保贷款2亿元以上，城镇新增就业1万人以上。

　　加快保障房建设。按照好房子、好环境、好服务的标准，加强保障房建设和管理。完善锦绣新城配套设施，加快城中村、成片棚户区和国有工矿区、城镇铁路沿线棚户区改造，全面完成地质灾害点群众避险搬迁三年计划任务。新开工保障房1.1万套，竣工1.3万套，2.4万人入住新居。全面开展房屋安全检查。

　　推进城乡社保体系建设。将企业退休人员基本养老金水平提高10%。城乡居民社会养老保险参保率保持在98%以上，城镇基本医疗保险参保率稳定在95%以上。城乡居民医疗保障政府补助水平提高到人均350元。提高最低工资和失业保险金标准。建设16个城市社区服务体系项目、28个农村社区服务中心。实施精准扶贫，年内完成1.79万农村贫困人口脱贫和5000人移民搬迁任务。推进城乡养老服务体系建设，大力发展妇女儿童事业，加强未成年人保护。做好困难家庭、残疾人、城市“三无”人员社会保障工作，支持慈善事业发展，让每一个身处困境者都能得到社会关爱和温暖。

　　办好人民满意的教育。实施学前教育分类管理，提升办园水平。开展标准化中小学创建。试行“大学区”管理模式，着力解决择校热、大班额问题。坚持立德树人，推进素质教育，减轻中小学生课业负担。推进“双高双普”创建工作。支持引导民办教育健康发展。通过国家二类城市语言文字达标验收。完成400名困难大学生资助、6000名困难大学生生源地助学贷款。推进铜川职业技术学院二期工程建设。

　　提高人民健康水平。优化医疗资源配置，加快市人民医院新区二期工程建设，建成市中医医院新区项目，逐步改善矿区和乡村医疗设施条件。提高医务人员医德医技水平，构建和谐医患关系。实施全省区域卫生信息化试点项目，建成全市中心卫生院远程会诊系统。扩大基本药物制度实施范围。加快中医药健康服务能力建设。继续抓好重特大疾病医疗救助工作。实施公共卫生服务项目，加快卫生应急体系建设，提高重大传染病、慢性病和地方病、重性精神疾病防治能力。

　　推进文化惠民工程。培育和践行社会主义核心价值观，加强公民道德和精神文明建设，创建文明城市。扩大政府购买公共文化服务范围，促进基本公共文化服务标准化均等化。加大文化设施投入，启动铜川体育中心、文化艺术中心规划建设，建成照金镇、黄堡镇综合文体中心，建成广播电视户户通标准化乡镇服务站13个、村级农民体育健身工程60个。

　　加强和创新社会治理。深入开展平安铜川、法治铜川建设，推进普法依法治理工作。时刻绷紧安全生产这根弦，强化红线意识，严格落实责任，推进安全发展，坚决遏制重特大安全事故发生。强化食品药品领域的安全检查，用最严谨的标准、最严格的监管、最严厉的处罚、最严肃的问责，保障“舌尖上的安全”。加强防灾预警体系建设，提高地质、气象、地震灾害防御能力。加强公安基层队伍和基层基础建设，坚持“大巡防”和“严打整治”不动摇，完善立体化社会治安防控体系，打造三秦安全城市。认真开展矛盾纠纷排查调处工作，重视社会舆情的研判、引导和处置，实行网上受理信访制度，上网听民意、下网解民忧，及时就地解决群众合法合理诉求。

　　各位代表，让人民群众过上美好幸福的生活，是政府一切工作的出发点和落脚点。我们将竭诚尽力办好民生实事，让发展成果更多更公平惠及百姓，使全市人民体面劳动、快乐生活！

　　（六）以经济体制改革为重点，全面推进改革开放

　　推进行政体制改革。加大支持区县一切有利于发展的改革创新举措，区县富则铜川强。加快转变政府职能，继续取消和下放市级行政审批事项，清理行政事业性收费，全面落实工商注册制度便利化政策。完成市、区县政府机构改革，加快推进事业单位分类改革。加强机构编制管理，制定2014－2017年控编减编方案。制定新一轮乡镇机构改革意见。启动公务用车制度改革。

　　深化经济体制改革。积极引入非公有资本参与国有企业产权改造，大力发展混合所有制经济。认真落实促进非公经济发展的政策措施，制定非公有制企业进入公用事业经营领域等扶持办法。非公经济增加值占生产总值比重达到49%。推进资源性产品价格改革，建立健全居民用水、用电、用气阶梯价格制度。推进土地经营权流转，两年内完成农村土地承包经营权确权登记颁证工作。探索建立城乡统一的建设用地市场。深化财税体制改革，推进资源税改革，扩大“营改增”试点，逐步建立重点镇和旅游名镇实体财政体制。规范政府债务管理，建立风险预警和偿还机制。

　　加快社会领域改革。深化收入分配制度改革，建立城乡居民收入与经济增长联动机制。加快社会征信体系建设，优化金融生态环境，建立失信者“黑名单”制度，让失信者受损，让守信者得益。逐步取消中小学校行政级别，深化高中招生制度改革。推行城乡居民大病保险，做好城镇基本医疗保险省内异地就医即时结算工作。深化县级公立医院改革。实施“单独两孩”政策。健全农村“三留守”人员关爱服务体系。深化殡葬改革。制定农村宅基地管理办法，依法保护被征地拆迁群众的合法权益。

　　发展开放型经济。以西安建设丝绸之路经济带新起点为契机，主动融入，差异化发展，推进西铜同城化。通过“负面清单”管理模式、“鼓励清单”服务模式、“权力清单”审改模式，以“权力”换“能力”，以权力的让渡实现政府服务能力的提升，优化发展环境。积极培育外贸骨干企业，扩大苹果、陶瓷、中医药、机电设备等产品出口，全年实现外贸进出口总额2000万美元。

　　四、加强法治政府和服务型政府建设

　　把学习贯彻党的十八届三中全会精神作为重大政治任务，扎实开展党的群众路线教育实践活动，加强政府自身建设，推进依法行政、科学施政、廉洁从政，着力增强政府执行力和公信力。

　　坚持依法行政。用法治思维和法治方式履行职责，自觉接受市人大工作监督、法律监督和市政协民主监督，认真听取各民主党派、工商联、无党派人士和人民团体的意见建议。健全领导干部学法制度和市政府法律顾问制度。完善行政决策规则，把公众参与、专家论证、风险评估、合法性审查和集体讨论决定作为重大决策的必经程序，增强公共政策制定透明度和公众参与度。加大公务员培训力度，全面提高依法行政能力和为民服务水平。

　　推进政务公开。信息公开是政府的义务，申请公开是公民的权利。完善政务和各领域办事公开制度，健全政府信息发布制度，及时全面准确公开群众普遍关心的政府信息。各级政府预算和决算向社会公开，部门预算公开到基本支出和项目支出。所有财政拨款安排的“三公”经费都要详细公开，细化说明“三公”经费增减变化原因等信息。健全百姓问政制度，搞好公民代表进政府、大学生暑期到政府机关见习活动，完善公众代表列席政务会议制度，拓宽公众意见反映和诉求渠道。做好网民留言办理和政务微博发布工作，及时回应民情民意民声，接受人民群众和新闻舆论监督。

　　改进工作作风。认真贯彻中央“八项规定”和省、市委的实施意见，深化“为民服务零距离”活动，自觉践行“三严三实”，锤炼过硬作风。精简会议活动，改进会风文风。开展“抓落实、促发展”专项执法监察活动，强化行政问责和效能监察，启动实施末位查究制，持续治理“庸懒散”，一切从严管理，严字当头才有好作风。

　　加强廉政建设。严格执行党政机关厉行节约反对浪费条例和国务院“约法三章”，坚持过紧日子。政府性楼堂馆所一律不得新建和改扩建，财政供养人员总量只减不增，“三公”经费只减不增。对公款出国、公款购车用车更要严加管理。加大部门预算执行和领导干部经济责任审计力度，真正替老百姓看管好“钱袋子”。深入开展损害群众利益突出问题专项治理，加大查办案件工作力度，切实做到干部清正、政府清廉、政治清明。

　　各位代表！最好的落实是一抓到底，最大的公信是说到做到。让我们以党的十八大和十八届三中全会精神为指引，在市委的坚强领导下，埋头苦干，奋勇争先，以更大的智慧和力量，扎扎实实做好各项工作，2014年要比2013年干得更精彩，为全面转型发展打下更坚实的基础！
